# Age-dependent prevalence of the Notch of Harty (pseudolesion of the tibial plafond) in children, adolescents, and young adults

**DOI:** 10.1186/s13244-025-02126-y

**Published:** 2025-11-09

**Authors:** Pauline Maria Hiller, Maximilian Schmalfuss, Lara Diedrichsen, Tobias Johannes Dietrich, Simon Wildermuth, Stephan Waelti, Anna Falkowski, Nicole Graf, Tim Steffen Fischer

**Affiliations:** 1 Division of Radiology and Nuclear Medicine, HOCH Health, Cantonal Hospital St. Gallen, St. Gallen, Switzerland; 2 Faculty of Medicine, University of Zurich, Zurich, Switzerland; 3 School of Medicine, University of St. Gallen, St.Gallen, Switzerland; 4 Division of Radiology and Nuclear Medicine, Cantonal Hospital Winterthur, Winterthur, Switzerland; 5 Clinical Trials Unit, Cantonal Hospital St. Gallen, St. Gallen, Switzerland

**Keywords:** Magnetic resonance imaging, Ankle joint, Pseudolesion, Developmental variant

## Abstract

**Objectives:**

The notch of Harty (NOH) is a pseudolesion located at the distal tibial plafond that can mimic a clinically significant osteochondral lesion. Reported prevalence in an adult population ranged between 25% and 45%, but the prevalence in younger age groups is unknown. In this study, we assessed the age-dependent prevalence of the NOH in a young population to investigate potential remodeling over time.

**Materials and methods:**

A retrospective analysis of a single-center database included 895 ankle MRIs of patients aged 6–25 years between 2018 and 2022. Cases with significant artifacts or pathological conditions were excluded. The NOH was categorized into types 1 (fluid-filled) and 2 (cartilage-filled), and measurements were taken for mediolateral, craniocaudal, and anteroposterior dimensions. Logistic and linear regression models with restricted cubic splines assessed age-related trends. Radiographic sensitivity for NOH detection was evaluated in 252 radiographs with MRI-confirmed NOH as a secondary endpoint.

**Results:**

The overall prevalence of NOH in this study population was 47.1% (14.9% type 1 and 31.6% type 2). Type 1 prevalence peaked at age 8 and declined into adulthood, while type 2 prevalence peaked at age 14 and stabilized through adolescence. The NOH size increased with age until 16 years and subsequently decreased. Radiographic sensitivity for detecting NOH was 51.2%.

**Conclusion:**

The NOH is more common in younger individuals, with prevalence and size decreasing with age. This supports its classification as a developmental variant characterized by age-related remodeling.

**Critical relevance statement:**

This study provides novel insights into the age-dependent prevalence and remodeling of the NOH, enhancing diagnostic precision and improving differentiation between developmental variants and pathological findings in clinical radiology.

**Key Points:**

- The study investigates the age-dependent prevalence of the NOH.
- Results show developmental remodeling patterns with prevalence peaking in younger individuals.
- Data support the theory of NOH being a developmental variant.

**Graphical Abstract:**

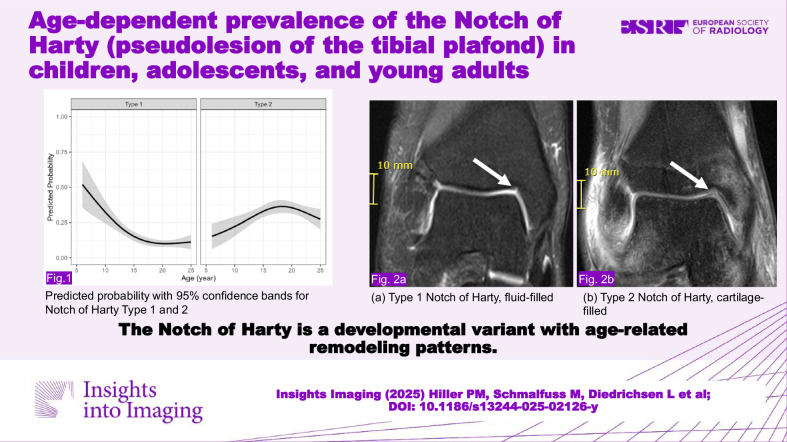

## Introduction

MRI is the preferred method for joint evaluation due to its superior soft tissue contrast [1–3]. In younger individuals, ankle MRI is mostly performed due to trauma, whereas the reason for imaging shifts towards degenerative disease in older individuals [4]. Proper evaluation of ankle MR images requires the radiologist to understand complex anatomy and the key disorders [3, 5], especially in the pediatric population, where developmental features must not be confused with pathological findings [6]. One well-known anatomical variant that may be mistaken for a cartilage defect is the supraacetabular fossa (SAF), located in the acetabular roof near the 12 o’clock position [7, 8]. Other variants include the pseudodefect of the capitulum of the elbow [9].

The notch of Harty (NOH) is an anatomical variant of the distal tibial plafond on MRI and needs to be distinguished from a traumatic osteochondral lesion. Described initially in 1988 by Harty as a notch of 3–5 mm [5], it is located at the osteochondral contour at the anteromedial margin of the tibial plafond. The MRI study of Boutin et al was the first to analyze the frequency and characteristics of the NOH; in 106 patients, it was present in 48 (45%) cases. These lesions typically appeared small and shallow, lacking trauma-associated subchondral changes, and measured 5–10 mm in width and 5.4 mm in length [5]. In a more recent study, Arndt et al found a prevalence of the NOH of 25% [10].

Pseudolesions tend to show different signal alterations, Dietrich et al categorized the SAF into two types based on MRI findings: type 1 (fluid-filled), which is more common in younger patients, and type 2 (cartilage-filled), suggesting that SAF may remodel with age [8]. Vaeth et al supported this hypothesis, observing that SAF prevalence and size decreased with age, indicating it may be a developmental variant [7].

To date, the age-dependent prevalence of NOH remains unreported. In this study, we investigated the age-dependent prevalence and the categorization of NOH in children, adolescents, and young adults. A deeper understanding of the development of NOH could enhance diagnostic accuracy, helping radiologists to avoid pitfalls and determine the correct diagnosis.

## Materials and methods

This retrospective study was approved by the Ethics Committee of Eastern Switzerland (Ethikkommission Ostschweiz, EKOS; BASEC ID 2023-01808) and was conducted using the digital database of our hospital system. Informed consent was obtained from all included individuals.

### Participant inclusion

The review focused on MRI examinations of the ankle, including MR arthrography, non-contrast MRIs, and MRIs with intravenous contrast, performed between 2018 and 2020. We included patients between 0 and 25; however, the youngest patient who underwent MRI imaging of the ankle with our selection criteria was 6 years old.

The primary retrospective review of our institutional database revealed 934 MR examinations of the ankle. Cases were excluded due to clinical conditions such as fractures of the talus, malleolus medialis, or distal tibia (*n* = 13), congenital deformities such as clubfoot (*n* = 2), or osteochondral lesions of the talus (*n* = 19). In cases of multiple examinations per patient, only the first scan was retained (*n* = 15). Additional exclusions were made for technical reasons, including metal artifacts (*n* = 5), absence of coronal imaging (*n* = 9), missing image data (*n* = 2), or severe motion artifacts (*n* = 8). After applying all exclusion criteria, a total of 895 MRI examinations from patients aged between 6 years and 25 years were included in the final analysis.

### MR imaging

All patients underwent MRI of the ankle joint using either a 1.5- or 3-Tesla MRI scanner in our institutions (Magnetom Skyra, Magnetom Skyrafit, Magnetom Vida, Magnetom Avantofit, and Magnetom Area, Siemens scanners, Siemens Healthineers, Erlangen, Germany). Standardized imaging protocols were primarily used, with adjustments made based on clinical indications such as the need for tarsal tunnel assessment or contrast enhancement. The standard protocol included proton density (PD) and T1-weighted turbo spin echo (TSE) sequences with and without fat suppression (FS), covering coronal, sagittal, and transversal planes. A standard protocol for ankle MRI is shown in Table 1.

**Table 1:** Example of standard scanning parameters for ankle MRI in our clinic using a 3-Tesla MRI scanner (Siemens 3 T Skyra)

Sequence	Plane	Field of view in mm	Echo time in ms	Repetition time in ms	Flip angle in °	Slice thickness in mm
PD TSE	Transverse	120	22	2200	140	3
PD TSE FS	Sagittal	150	41	2500	150	3
PD TSE FS	Coronal	100	25	2900	150	3
T1 TSE	Coronal	100	15	490	140	3

*mm* millimeters, *ms* milliseconds, *FS* fat-suppressed, *PD* proton density

### MR image evaluation

A senior radiology resident (M.S.) with four years of experience, a medical student conducting her thesis (P.M.H.), and another medical student (L.D.) reviewed all cases using the institution’s routine clinical workstations and PACS software (Dedalus DeepUnity Diagnost 1.1.0.1, Germany). All obtained sequences were reviewed to assess the presence and type of NOH. The primary sequences, uniformly available in all evaluated MRI examinations for lesion measurement, included T1-weighted TSE (T1 TSE) coronal, PD fat-suppressed TSE (PD TSE FS) coronal, and turbo inversion recovery magnitude (TIRM) sagittal sequences. Additional sequences may have varied depending on the examination. MRI examinations were reviewed individually and evaluated for the presence or absence of the NOH. If present, the NOH was scored as type 1 if a fluid-like signal was visible, and type 2 if a cartilage-like signal was visible. NOH dimensions were measured using coronal PD-TSE with fat suppression and sagittal TIRM FS sequences, with measurements taken from cortex to cortex using the PACS toolbox in the anterior-posterior, medial-lateral, and cranial-caudal dimensions, and reported in millimeters. Discordant assessments (presence/absence or type 1/type 2) were resolved by a senior radiologist (T.S.F.) with 8 years of experience, including three years dedicated to musculoskeletal imaging.

### Radiograph evaluation

As a secondary endpoint, the sensitivity of radiography for NOH detection was evaluated. The radiology database was checked for radiographs of the ankle taken within six months prior to or after an MRI examination with a visible NOH on MRI. All eligible radiographs (*n* = 252) were evaluated for visible NOH by a senior radiology resident (M.S.) with four years of experience and a medical student (P.M.H.).

### Statistical analysis

Analyses were performed by a statistician (N.G.). All analyses were performed in the R programming language (version 4.2.2) [11]. The package “tableone” [12] was used to compute descriptive statistics. The package “psych” [13] was used to compute Cohen’s kappa. The package “rms” [14] was used to add restricted cubic splines. The package “ggplot2” [15] was used to plot the figures.

Reliability of the assessments was measured using Cohen’s kappa for nominal data. Logistic regression models were employed to evaluate the influence of age on the presence of type 1 and type 2 Notches of Harty, incorporating restricted cubic splines with three knots to relax the linearity assumption. Similarly, linear regression models with restricted cubic splines were used to assess the influence of age on the dimensions of the NOH. Assumptions of linearity, normality, and homoskedasticity were verified through residual plots. Sensitivity and exact 95% confidence intervals (CI) for binomial probabilities were calculated for radiographs.

For missing values, an available data analysis approach was used. While age and the presence of NOH were available for all 895 patients, the three dimensions of the NOH were measured in 401 cases by the resident (M.S.) and in 340 cases by both the resident and the medical student (P.M.H.). The sensitivity of radiography was assessed using 252 images where NOH was confirmed by MRI.

## Results

**Fig. 1:**
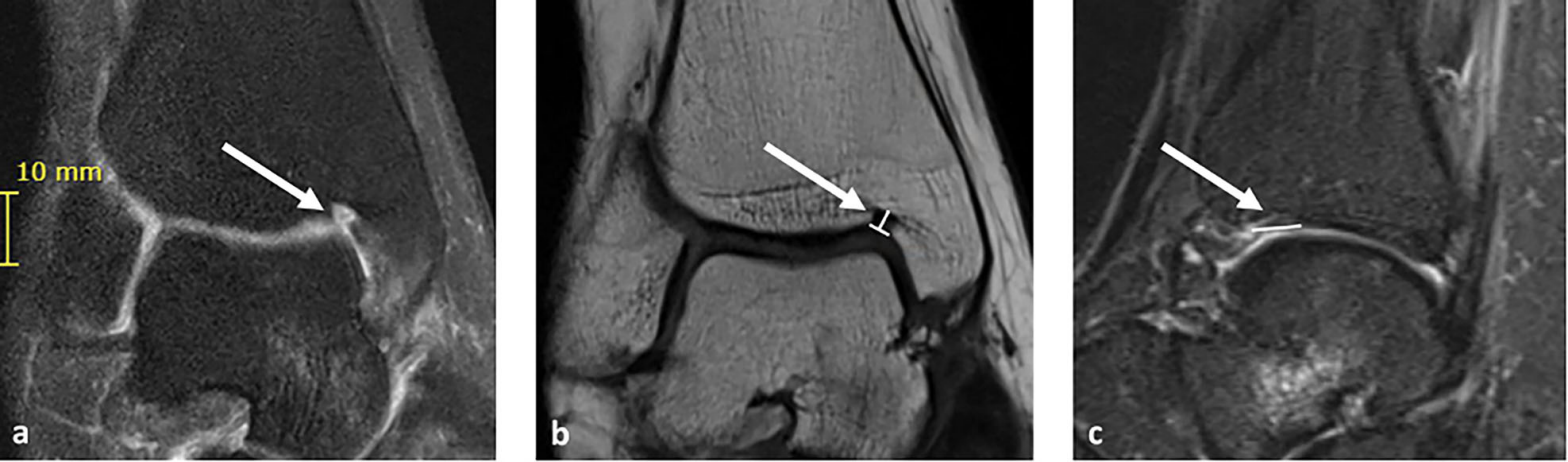
MRI of the right ankle in a 15-year-old female participant demonstrating a type 1 NOH (white arrows). **a** Coronal proton density-weighted turbo spin echo with fat suppression, (**b**) coronal T1-weighted turbo spin echo (TSE) with calipers measuring height and width, and (**c**) sagittal turbo inversion recovery magnitude (TIRM) sequence with fat suppression and contrast with calipers measuring depth. The NOH dimensions are 3 mm (height), 3 mm (width), and 6 mm (depth)

**Fig. 2:**
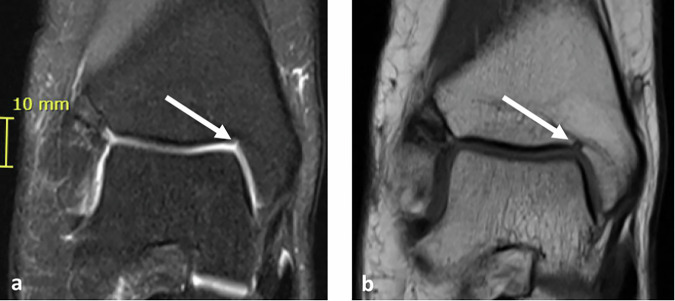
MRI of the right ankle in a 16-year-old female with a small and shallow type 1 NOH (white arrows). Dimensions of the notch are 2 mm (height) 2 mm (width) and 4 mm (depth). **a** Proton density weighted with fat suppression and (**b**) T1 weighted coronal sections

**Fig. 3:**
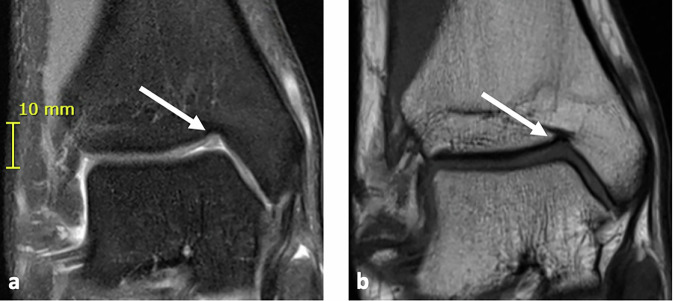
MRI of the right ankle in a 19-year-old male demonstrating a type 2 NOH (white arrows) measuring 4 mm (heigh) 2 mm (width) and 5 mm (depth). **a** Coronal proton density-weighted turbo spin echo with fat suppression and (**b**) coronal TSE illustrates the cartilage-filled characteristics of the type 2 NOH

A total of 895 patients were included in the analysis. The mean age was 17.9 years, ranging between 6 to 25 years. The NOH was present in 47.1% of the study population. Imaging examples for NOH type 1 and type 2 are given in Figs. 1 and 2 for NOH type 1 and Fig. 3 for NOH type 2. A total of 133 (14.9%) presented with NOH type 1 and 283 (31.6%) with NOH type 2. The mean (SD) dimensions of the NOH were measured as 6.6 mm (2.5) mediolaterally, 2.7 mm (0.9) craniocaudally, and 5.4 mm (1.9) anteroposterior (Table 2).

**Table 2:** Description of study population, including age, presence of NOH, and its dimensions

Age (mean in years (SD))	17.9 (4.5)
Decision specialist	
NOH not present (%)	479/895 (53.5)
NOH type 1 (%)	133/895 (14.9)
NOH type 2 (%)	283/895 (31.6)
Mediolateral (mean in mm (SD))	6.6 (2.5)
Craniocaudal (mean in mm (SD))	2.7 (0.9)
Anteroposterior (mean in mm (SD))	5.4 (1.9)

*SD* standard deviation, *mm* millimeters

**Fig. 4:**
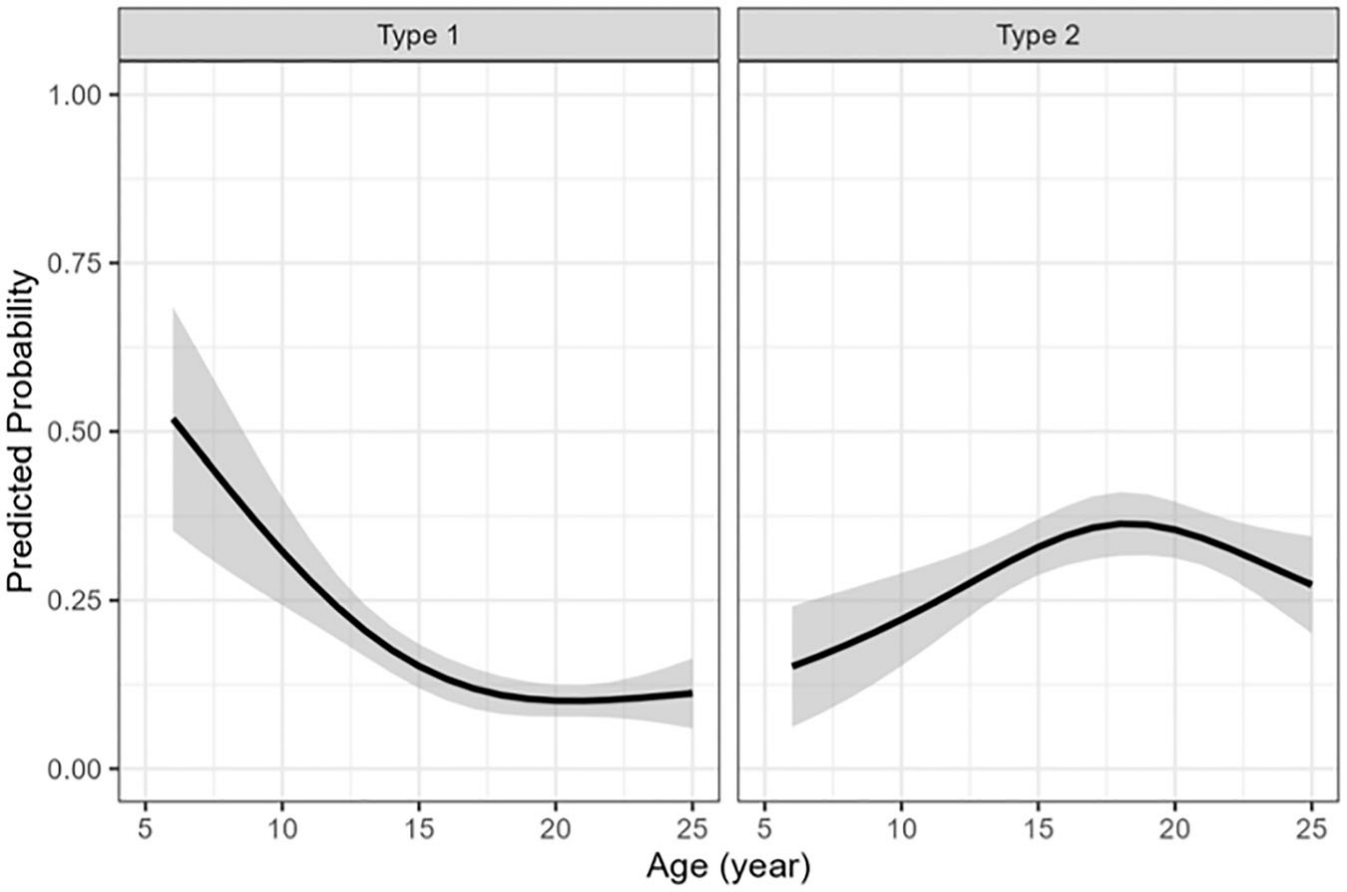
Predicted probability with 95% confidence bands for Notch of Harty type 1 and type 2

The prevalence of NOH varied with age for both types 1 and 2 (Table 3a, b). To estimate the age-dependent probabilities of detecting NOH types 1 and 2, predicted probabilities derived from the logistic regression model indicated that NOH type 1 prevalence sharply declined with age, stabilizing around 10% in adults. For type 2, prevalence increased with age, peaking around 18 years before gradually decreasing as listed in Table 3a, b. Larger CIs were observed in younger ages, reflecting greater variability in these predictions. These age-related probability trends are presented in Fig. 4, with 95% confidence bands.

**Table 3:** a: Number of patients (*n*), as well as number (Notch) and rate (rate) of NOH type 1 per age group, and b: number of patients (*n*), as well as number (Notch) and rate (rate) of NOH type 2 per age group

Age	*n*	Notch	Rate (%)
**a**			
6	1	0	0.0
7	3	1	33.3
8	10	7	70.0
9	19	7	36.8
10	31	11	35.5
11	26	5	19.2
12	27	6	22.2
13	42	8	19.0
14	58	12	20.7
15	61	7	11.5
16	69	4	5.8
17	70	10	14.3
18	74	12	16.2
19	68	10	14.7
20	44	6	13.6
21	58	4	6.9
22	66	3	4.5
23	46	5	10.9
24	68	10	14.7
25	54	5	9.3
**b**			
6	1	0	0.0
7	3	0	0.0
8	10	0	0.0
9	19	2	10.53
10	31	5	16.13
11	26	9	34.62
12	27	7	25.93
13	42	19	45.24
14	58	19	32.76
15	61	26	42.62
16	69	23	33.33
17	70	18	25.71
18	74	22	29.723
19	68	25	36.76
20	44	17	38.64
21	58	21	36.21
22	66	22	33.33
23	46	10	21.74
24	68	23	33.82
25	54	15	27.78

*n* number of patients

The association between age and NOH presence was statistically significant for both types: type 1 (*p* < 0.001) and type 2 (*p* = 0.013), as determined by the Wald test comparing models with and without the age variable. These findings support a non-linear, age-dependent remodeling process underlying the appearance of the NOH.

**Fig. 5:**
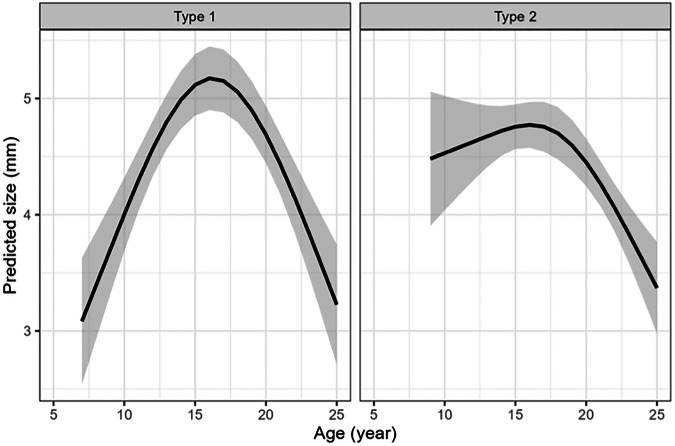
Predicted size of Notch of Harty types 1 and 2 with 95% confidence bands

The size of NOH was analyzed by calculating its volume as the product of the mediolateral, craniocaudal, and anteroposterior dimensions. Subsequently, the third root was taken to normalize the data. For both NOH types, size increased with age until around 16 years, after which it decreased. Notably, type 1 lesions started with smaller sizes and peaked at higher values than type 2 lesions (Fig. 5).

All 895 images were rated by the resident and both medical students (P.M.H. and L.D.). Cohen’s kappa was 0.87 (95% CI: 0.84–0.90) for the resident, 0.80 (95% CI: 0.76–0.83) for the doctoral student, and 0.84 (95% CI: 0.81–0.87) for the master's student. Thus, agreement with the specialist was substantial (P.M.H.) to almost perfect (M.S. and L.D.) [16]. Inter-rater reliability for NOH measurements was assessed using the intraclass correlation coefficient (ICC) between the specialist and the resident. The ICC demonstrated moderate agreement for mediolateral measurements (0.557, 95% CI: 0.318–0.702) and poor agreement for craniocaudal (0.339, 95% CI: −0.24 to 0.431) and anteroposterior (0.292, 95% CI: 0.174–0.399) dimensions.

As a secondary endpoint, radiograph sensitivity was assessed; of the 252 cases where NOH was confirmed by MRI, 129 were also detected on radiograph, resulting in a sensitivity of 51.2% (95% CI: 44.8–57.5%).

## Discussion

This study demonstrated that both the prevalence and size of NOH are age-dependent in this population. The NOH was most frequently observed between ages 8 and 15, with its prevalence decreasing in older age groups, which indicates that the NOH is a developmental variant. This anatomical variant or pseudolesion of the distal tibial plafond on MRI needs to be distinguished from a traumatic osteochondral lesion. Boutin et al reported that NOH was present in 45% of patients within a smaller and older study population (mean age 44.5 years) [5]. A more recent study by Arndt et al analyzed a larger study population of 2428 patients and reported a prevalence of 25%, though specific details regarding the age distribution were not provided [10].

Our study focused on a younger population (6–25 years), revealing an overall NOH prevalence of 47.1%, showing a decline towards the age of 25. This supports the hypothesis that NOH is a developmental variant that undergoes age-related remodeling: The NOH does not fully disappear with age, but the prevalence instead stabilizes at a lower, yet persistent rate. The lower prevalence among older individuals in our cohort corresponds with the 25% prevalence reported by Arndt et al [10]. A variation in prevalence, for example, a second peak in older adults, cannot be entirely excluded, but our data, and existing forensic knowledge of skeletal maturation, make this unlikely [7]. Differences in imaging protocols, measurement criteria, and patient selection may also account for different overall prevalence rates across studies.

In 2012, based on imaging, Dietrich et al categorized the SAF into two different types, with type 1 being fluid-filled and type 2 being cartilage-filled. The assumption was made that SAF may undergo a remodeling with increasing age, eventually developing into SAF type 2 [8]. Later, Vaeth et al supported this hypothesis with their finding that SAF is more frequent in adolescents and tends to decrease in prevalence and size with increasing age, supporting the theory that SAF is a developmental variant [7]. Similarly, Dietrich et al demonstrated an age-related transformation of the superior acetabular roof notch (SARN) from a fluid-like to a fat-like appearance on MRI, with the prevalence of type 1 SARN decreasing and type 2 SARN increasing during adolescence [17]. Age-related changes in osteochondral pseudolesions have been observed in various joints, as highlighted by Djebbar et al, who reported such changes in the glenoid bare spot [18].

**Fig. 6:**
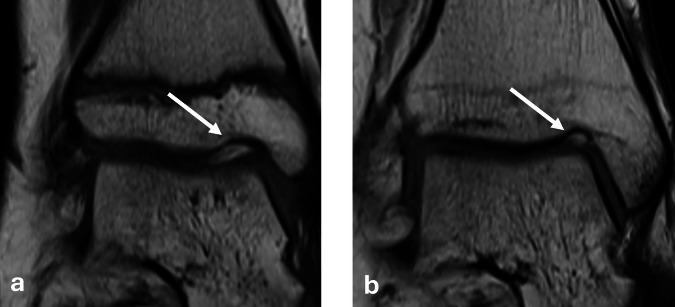
Coronal TSE MR images of the right ankle in a 13-year-old female patient at baseline (**a**) and after 7 years (**b**). White arrows indicate the Notch of Harty. The follow-up shows a clear morphological transformation over time, providing visual support for an age-related remodeling process

In this study, the prevalence of NOH shows a similar age-related pattern for both types, with type 1 (fluid-filled) peaking around age 8 and declining steadily into adulthood, while type 2 (cartilage-filled) peaks around age 14 and remains moderate through late adolescence. This distribution suggests that NOH type 1 may gradually develop into type 2 with age, matching the findings of previous research [7, 8, 17]. However, because of the smaller sample sizes among younger age groups, trends for the youngest ages are less certain. Figure 6 illustrates a follow-up case of NOH development over time. The patient was 13 years old at baseline, with a 7-year interval between scans. The visible morphological changes further support the hypothesis of an age-dependent evolution of the NOH. However, prospective longitudinal studies may be conducted to directly observe the evolution of the NOH.

A key differential diagnosis of the NOH is the osteochondral lesion. While our study did not aim to compare these entities directly, distinction is essential to avoid misinterpretation. NOH typically appears as a shallow, corticated, and well-defined concavity without adjacent bone marrow edema, cartilage disruption, or subchondral cysts [5, 10]. In contrast, osteochondral lesions are often irregular, involve subchondral and cartilage defects, and are frequently associated with surrounding edema or cystic change [2, 19].

Boutin et al found that the NOH had a mediolateral width of 5–10 mm and an anteroposterior length of 5.4 mm, with depth described as small and shallow [5]. In this study, the mean dimensions of the NOH were 6.6 mm mediolaterally, 2.7 mm craniocaudally, and 5.4 mm anteroposteriorly. A volume-based approach showed that for both types of NOH, size increased with age until around 16 years, after which it decreased (Fig. 5). Interestingly, type 1 lesions initially had smaller sizes but reached higher peak values than type 2 lesions, which further supports the theory of an age-dependent remodeling of the NOH. Size measurements were conducted by two readers, and the ICC was calculated to assess inter-rater reliability. While agreement was moderate for mediolateral dimensions, it was poor for craniocaudal and anteroposterior measurements. Similar discrepancies in measurement reliability have been reported in other musculoskeletal MRI studies, where factors such as slice thickness, motion artifacts, and anatomical variability can influence reproducibility [20]. Despite suboptimal agreement, we believe that size measurements, especially when combined with the evaluation of NOH presence, support our developmental theory and are therefore justified. This is further supported by existing data, which also indicates that non-traumatic osteochondral lesions change in an age-dependent manner over time [7, 17, 18].

While skeletal growth may contribute to changes in NOH dimensions, the non-linear trend observed in our study—an initial increase in size during early adolescence followed by a subsequent decline—is not consistent with proportional growth alone. Rather, this pattern supports the interpretation of a developmental remodeling process intrinsic to the NOH. Similar transient behavior has been described in other anatomical variants, such as the SAF [7] and the SARN [17], where size and signal characteristics change with age. In our cohort, the statistically significant association between age and lesion dimensions, along with the shift from type 1 to type 2 morphology, further reinforces this interpretation.

The sensitivity of radiography was assessed as a secondary endpoint, revealing limited effectiveness in detecting NOH. Of the 252 cases where MRI confirmed the presence of NOH, 129 were also visible on radiographs, yielding a sensitivity of 51.2%, which makes radiography not the modality of choice for NOH detection. These findings also align with expectations, as it has been demonstrated that conventional radiographs have only moderate sensitivity for detecting osteochondral defects, whereas computed tomography (CT) and MRI are more accurate modalities for evaluation of these [2, 21].

Limitations of this study include its retrospective design, which may introduce bias and limit causal interpretations, as well as its single-center setting, which may limit generalizability. Moreover, clinical correlation was limited, potential symptoms or functional impairments, as well as follow-up information, were not consistently available. The uneven age distribution, particularly the limited number of patients aged 6–8 years, represents an important limitation, as this age group showed the highest predicted prevalence of type 1 NOH. Given the small sample size (*n* = 8), our findings in this subgroup should be interpreted with caution. Future studies with dedicated pediatric cohorts are needed to validate these findings in younger populations. Inter-rater reliability was measured, but differences in observer experience may have influenced diagnostic consistency. Size measurements showed suboptimal agreement, particularly for craniocaudal and anteroposterior dimensions. Lesion size may in part reflect general skeletal growth. Due to our methodological approach, absolute lesion dimensions were used, and normalization for skeletal size was not performed. Therefore, a contribution of proportional growth to the observed size changes cannot be fully excluded, despite this, we believe the measurements support the developmental theory of NOH. Cartilage-sensitive sensitive-Gradient-Recalled Echo sequences, which may have provided additional insight into the structure of NOH Type II and its distinction from osteochondral lesions [22, 23], have not been uniformly available. This was due to the retrospective nature of the study, as imaging protocols slightly varied depending on the clinical indication. Radiographic evaluations were limited to MRI-confirmed NOH cases, and assessments were not blinded to age, posing a potential observer bias. Lastly, while our study focused on younger individuals, a secondary NOH peak in older adults cannot be entirely ruled out, though it remains unlikely given forensic age diagnostic knowledge indicating complete skeletal maturation by age 25 [7]. Future studies should incorporate standardized measurement protocols and advanced imaging techniques, such as 3D acquisitions, to improve reliability.

In conclusion, our findings confirm that NOH is more prevalent in younger individuals. This data supports the theory that NOH is a developmental variant. The age-related pattern suggests that NOH changes as part of normal skeletal growth. Throughout skeletal maturation, it may progress from a fluid-filled type 1 to a cartilage-filled type 2 notch, potentially disappearing entirely. This progression may pause at any stage, reflecting individual variation in developmental patterns.

## Data Availability

The dataset analyzed during this study is available from the corresponding author on reasonable request.

## Abbreviations

CI: Confidence interval
FS: Fat-suppressed
MRI: Magnetic resonance imaging
NOH: Notch of Harty
PD: Proton density
SAF: Supraacetabular fossa
SARN: Superior acetabular roof notch
SD: Standard deviation
TIRM: Turbo inversion recovery magnitude
TSE: Turbo spin echo
